# Jatrophone: a cytotoxic macrocylic diterpene targeting PI3K/AKT/NF-κB pathway, inducing apoptosis and autophagy in resistant breast cancer cells

**DOI:** 10.1186/s12906-023-04113-6

**Published:** 2023-08-22

**Authors:** Khawlah Shari, Rania A. El Gedaily, Rasha M. Allam, Khaled M. Meselhy, Amal E. Khaleel, Essam Abdel-Sattar

**Affiliations:** 1 Pharmacognosy Department, Faculty of Pharmacy, Cairo University, Kasr El-Aini St, Cairo, 11562 Egypt; 2 Pharmacology Department, Medical Research Institute, National Research Centre, Dokki, Cairo, 12622 Egypt

**Keywords:** Jatrophone, *Jatropha spinosa*, Doxorubicin-resistant breast Cancer, Early apoptosis, PI3K/AKT/NF-κB, Autophagy, Migration/*β*

## Abstract

**Background:**

Breast cancer is a prevalent malignant tumor that affects women worldwide. The primary challenge in treating breast cancer is combating drug resistance, which contributes to relapse and metastasis. Jatrophone is a unique macrocyclic jatrophane diterpene found in various *Jatropha* and *Euphorbia* species. It possesses diverse biological and pharmacological activities, including anticancer activity. However, it is unclear whether jatrophone can overcome drug resistance in breast cancer.

**Methods:**

This study includes the investigation of the cytotoxicity of jatrophone on doxorubicin-resistant breast cancer cells (MCF-7^ADR^) and the underlying molecular mechanisms. The effects of jatrophone on cell viability were determined using the sulforhodamine B (SRB) assay, while flow cytometry was used to evaluate cell cycle progression, apoptosis, and autophagy. A scratch assay was conducted to observe cell migration, and western blotting was used to measure downstream protein levels (PI3K, AKT, and NF-κB). Unpaired Student’s t-tests were used for comparison between the two groups and the results were analyzed by one-way ANOVA with Tukey- Kremer post hoc test.

**Results:**

It was shown that jatrophone exhibited potent cytotoxic activity on MCF-7^ADR^ cells in a dose-dependent manner, with an IC_50_ value of 1.8 µM. It also significantly induced cell cycle S and G/M phase arrest. Interestingly, jatrophone induced both early and late apoptotic cell death, as well as autophagic cell death, with negligible necrosis. Furthermore, jatrophone treatment diminished the migration of MCF-7^ADR^ cells. At the molecular level, jatrophone treatment significantly down-regulated the expression levels of PI3K, AKT, and NF-κB. *β*.

**Conclusions:**

The results of the study suggest that jatrophone decreases the proliferation of MCF-7/ADR cells at a low micromolar concentration; induces cell cycle arrest; promotes apoptotic, and autophagic cell death; inhibits migration and EMT; and works on resistance by a mechanism involving the inhibition of the PI3K/Akt/ NF-κB pathway. These findings provide evidence of the potential of jatrophone to be a promising lead compound for targeting doxorubicin-resistant breast cancer cells and could be further investigated for its clinical application as a chemotherapy adjuvant.

**Supplementary Information:**

The online version contains supplementary material available at 10.1186/s12906-023-04113-6.

## Introduction

Breast cancer (BC) is posing a significant threat to women’s health as its incidence and mortality rates have increased significantly in recent years, affecting younger individuals [1]. In 2020, breast cancer surpassed lung cancer as the leading cause of cancer incidence globally, with 2.26 million new cases, accounting for about 11.7% of all new cancer cases [2]. Although surgery followed by chemotherapy or radiotherapy is a common treatment approach for breast cancer, drug resistance remains a significant obstacle, leading to tumor relapse and metastasis in 30–40% of patients [3]. As a result, novel strategies are required to overcome the developed drug resistance, which seems to be a big challenge in breast cancer treatment.

Plant-derived compounds have shown promise in attenuating drug resistance and exhibiting potent anticancer effects. Phytochemicals found in foods or traditional medicines are now being explored as a multi-therapy approach for cancer treatment [4–6]. Jatrophone (JA) is the first jatrophane type macrocyclic diterpene, isolated from several *Jatropha* species, with proven pharmacological activities, including anticancer activity [7]. *Jatropha* displayed a broad range of pharmacological activities including anticancer activity [8]. Nevertheless, a panel of cancer cell lines representing a range of cancers, including lymphocytic leukaemia, cervical, prostate, and colon cancer, were tested against various fractions of *Jatropha* species [9–12]. It should be noted that only a small number of studies on JA itself have demonstrated its anticancer activity in vitro against hepatocellular cancerous cells [13], glioma [14] and triple-negative breast cancer [15], and the mechanism of this activity is still elusive and undefined.

The aberrant activation of the phosphoinositide 3-kinase (PI3K)/protein kinase (AKT) signaling pathway is one of the signaling cascades that is most frequently activated in the etiology of breast cancer. The activated PI3K/Akt pathway can promote rapid cell growth and the development of multidrug resistance in tumor cells *via* dysregulate breast cancer cells’ survival, proliferation, apoptosis, and other processes [16]. Additionally, according to several studies, activated PI3K/Akt/NF-B encourages migration and triggers the epithelial-mesenchymal transition (EMT), which is crucial for the growth and metastasis of breast cancer [17–19]. Therefore, the PI3K/AKT/NF-κB signaling pathway is an important therapeutic target for treatment.

Autophagy deregulation can lead to increased tumorigenesis and resistance to several therapies in breast cancer [20–22]. Autophagy plays a double-edged sword role as it either promotes the survival of multi-drug resistance (MDR) tumors during starvation or leads to type II programmed cell death. Therefore, understanding its exact role in response to different treatments [23].

Jatrophone (JA) is a macrocyclic diterpene isolated from different *Jatropha* species [24, 25]., and its derivatives have shown multiple biological properties, including insulin inhibition, lymphocyte activation inhibition, tumor cell inhibition, molluscicidal activity, and gastroprotective benefits [26–28].

This study aimed to investigate the potential cytotoxic effect of JA on MCF-7^ADR^ cells and to understand the molecular mechanism involved in cell cycle arrest, apoptosis, and autophagy. Additionally, the anti-migratory effect of JA was determined, and the expression of PI3K/Akt/NF-κB, *β* was investigated.

## Materials and methods

### Plant material

The roots of *J. spinosa* were collected from Magbanah Mountain at Taiz governorate, Yemen, in February, 2020. The collection and handling of plant were in accordance with all the relevant guidelines. The plant was identified and authenticated by Agricultural Engineer Abd Alhabib Al-ghadasi in the Agriculture Research Institute of Yemen. Voucher specimen (PN-2-11-2022ÌÌ) was put in the herbarium of the Faculty of Pharmacy, Cairo University.

### Extraction and isolation

The powdered sample of *J. spinosa* root (1500 g) was extracted two times (7.5 L, each) with methanol (2 × 7.5 L) by maceration for 48 h. The extract was concentrated under reduced pressure to give a brown residue (125 g). The crude methanol extract (90 g) was suspended in water/methanol mixture (400 mL) followed by partitioning with dichloromethane (CH_2_Cl_2_, 5 × 300 mL) to give CH_2_Cl_2_ fraction (19 g) and water remain (65 g). The CH_2_Cl_2_ fraction (15 g) was subjected to chromatography on a Si gel column (70–230 mesh size) and eluted with EtOAc-n-hexane (0→100%, v/v) using a stepwise gradient mode. A total of 27 fractions (50 mL each) were collected. TLC monitoring using *p*-anisaldehyde/H_2_SO_4_ as spray reagent resulted in 15 combined fractions. Fraction **9** (**Fr-9**, 2.25 g) on chromatography using a Si gel column eluted with EtOAc-n-hexane (8:2) gave four fractions. Subfraction **3** on crystallization from methanol gave compound **1** (1.5 g). The isolated compound (**1**) was analyzed using NMR spectroscopy and was identified by comparison to data reported in the literature (See the results section).

### Methodology

#### Cell culture

Human doxorubicin resistant breast cancer cell line (MCF-^7ADR^) was obtained from Nawah Scientific Inc., (Mokatam, Cairo, Egypt) and cultured in DMEM (Dulbecco’s modified eagle’s medium), Gibco-Thermo Fisher Scientific, Waltham, MA, USA. The culture media were supplemented with 10% fetal bovine serum (FBS), and 100 units/mL penicillin/streptomycin (PS). The cells were passaged in a dampened atmosphere, 5% CO2 and at 37 ^0^ C.

#### Cytotoxicity assay

The cytotoxic activity of JA against MCF-7^ADR^ cells was assessed by sulphrhodamine B (SRB) assay. Cells were seeded in 96-well plates, approximately 5 × 10^**3**^/well, and treated with JA at a concentration of (100, 10, 1.0, 0.1, and 0.01 µM) for 72 h. After that, the media were discarded, and 150 µL of 10% trichloroacetic acid (TCA)/ well were added (Merck) for 1 h in a fridge. Then, the cells were washed with tap water three times. Afterward, 70 µL of SRB solution (0.4% w/v) (Sigma‐Aldrich) was added for 10 min in a dark place at room temperature. The cells were first rinsed with 1% acetic acid three times and then air-dried overnight. Next, 150 µL of 10 mM Tris Base was added to dissolve the protein-bound stain. Finally, the absorbance of the solution was measured at 540 nm using a FluoStar Omega microplate reader (BMG Labtec, Ortenberg, Germany). The dose-response curve of JA was analyzed using the Emax model, as described in a previous publication [29].

#### Cell cycle analysis

After 48 h of exposure to the pre-calculated IC_50_ value of jatrophone, MCF-7^ADR^ cells were extracted using trypsin. The cells were then fixed in ice-cold 60% ethanol in PBS at 40 ^o^C overnight after being washed twice in phosphate-buffered saline (PBS). Following PBS washing, cells were resuspended in 500 µL of propidium iodide (PI) with RNase staining buffer and incubated for 35 min before readings were obtained from the flow cytometer (ACEA NovocyteTM flow cytometer, ACEA Biosciences Inc. San Diego, California). Data were analyzed using ACEA Novo ExpressTM software (ACEA Biosciences Inc., San Diego, USA) and a minimum of 12,000 cells/events were obtained each sample [30].

#### Annexin V-FITC /PI apoptosis assay

Following the manufacturer’s instructions, the Annexin V-FITC/PI Apoptosis Detection Kit (BD Biosciences, San Diego, USA) was used to examine early/late apoptotic and necrotic cells. MCF-7**ADR** cells were treated with the pre-calculated IC_50_ value of jatrophone for 48 h, then collected with trypsin and rinsed with PBS (two times). Then, for 30 min at room temperature in the dark, cells were once more resuspended in 0.5 ml of binding buffer containing 5 µL of Annexin V-FITC and 5 µL PI. Within an hour of staining, cell apoptosis/necrosis was then detected using a flow cytometer (ACEA Biosciences Inc., San Diego, CA, USA) [31].

#### Autophagy analysis

MCF-7^**ADR**^ cells were treated with jatrophone for 48 h, trypsinzed, and washed twice with ice-cold PBS. The cells were then stained with 0.5 ml of acridine orange staining solution (1 µg /mL in PBS) for 30 min at room temperature in absence of light. Flow cytometric analysis was performed *via* ACEA Novocyte™ flow cytometer, and the fluorescent signals were analyzed *via* FL1 signal detector (488 nm excitation/530nm emission). Net fluorescent intensities (NFI) were calculated based on the analysis of 12,000 cell events [32].

#### Migration assay

To examine the effect of jatrophone on the migration of resistant breast cancer cells, a cell scratch assay was performed. MCF-7^ADR^ cells were seeded in a 6-well plate and cultured in the appropriate medium until they reached 95% confluency. The cells were then starved for 6 h in a serum-free medium. Scratches were created using a sterile 200-µL pipette tip, and the cells were rinsed twice with PBS to remove debris. The cells were then maintained in fresh media with or without jatrophone. Images were taken at 0, 24, 48, and 72 h using an inverted microscope (LABOMED model TCM 400, Labo America, Inc, USA) until the control wound completely closed and the wound widths were measured using ImageJ software [33].

#### Western blot assay

Cells were lysed with EDTA-free Protease Inhibitor in 10 mM Tris-HCl, 100 mM NaCl, 0.5% Triton X-100, pH 7.6. Protein concentration was assessed using the Pierce**™** BCA Protein Assay Kit (ThermoScientific). Following blocking with 5% non-fat milk for 2 h at room temperature, equal amounts of protein (35 µg) were subjected to 10% sodium dodecyl sulfate-polyacrylamide gel (SDS-PAGE) and then transferred to PVDF membranes (Millipore, Bedford, Massachusetts). Western blot analysis was conducted with primary antibodies against *β*, NF-kB p100/p52 Ab (Cell Signaling *Cat*. #, 8242T), Phospho-Akt (Ser473) (Cell Signaling *Cat*. #, 9271T), and PI3K (Cell Signaling *Cat*. #, 4249T),. HRP-conjugated secondary antibody (Cell Signaling, *Cat*. #,7074P2) was used to detect specific signals. Band intensities were analyzed by ChemiDoc™ imaging system with Image Lab™ software version 5.1 (Bio-Rad Laboratories Inc., Hercules, CA, USA). The protein expression level was normalized to β-actin (Cell Signaling *Cat*. #, 4970 S) [34].

### Statistical analysis

Data were displayed as mean ± SD. Unpaired Student’s t-tests were used for comparison between the two groups. Results were analyzed by one-way ANOVA with Tukey- Kremer post hoc test. All statistical analyses were done using GraphPad Prism™ software version 6.00 (GraphPad Software, Inc., La Jolla, CA, USA). Statistical significance was selected at P ≤ 0.05.

## Results

The MeOH extract of the roots of *J. spinosa* was subjected to chromatographic fractionation and separation on a Si gel column, which resulted in the isolation of a major macrocyclic diterpene (**1**), The identification of this compound was based on NMR analysis, and comparison with literature data [35, 36].


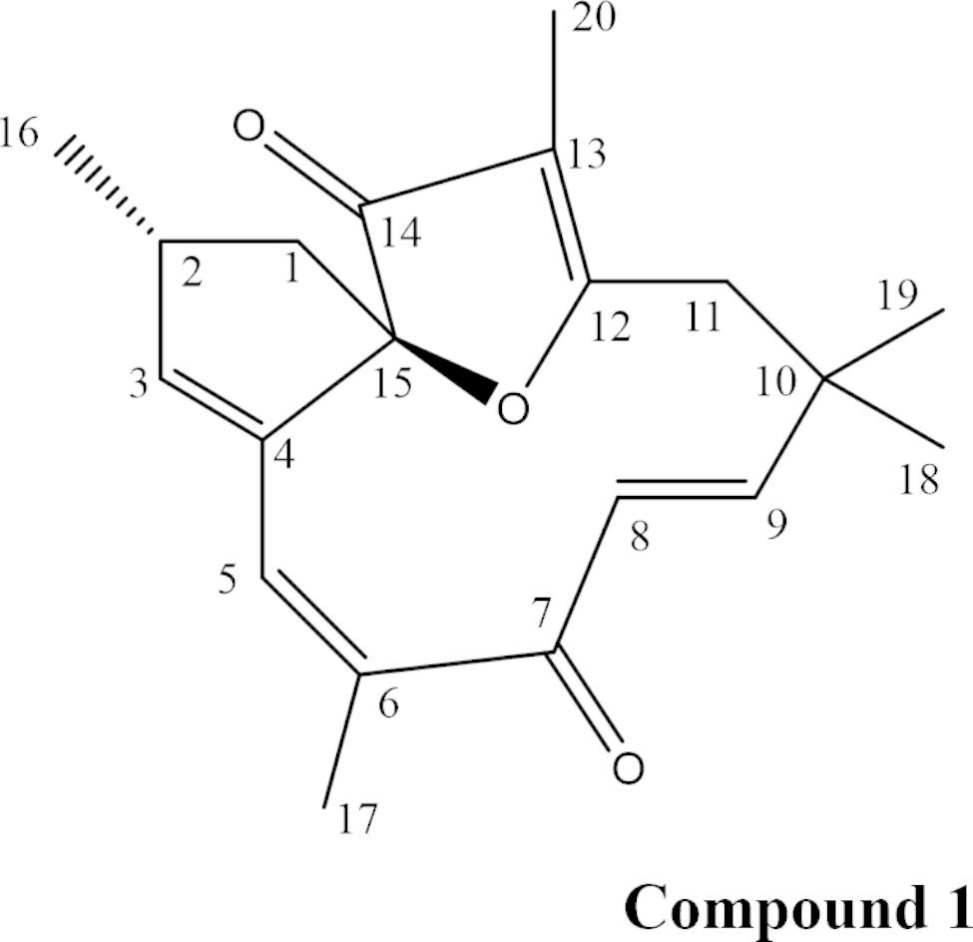


Compound **1** was isolated as colorless needles (1.5 g). The ^1^ H-NMR spectrum displayed signals for four vinylic proton at δ_H_ 6.44 (1 H, *d*, *J* = 16.3 Hz, H-9), 5.98 (1 H, *d*, *J* = 16.3 Hz,, H-8), 5.80 (2 H, *dt*, *J* = 8.4, 1.8 Hz, H-5, H-3), and five methyl protons at δ_H_ 1.87 (s), 1.74 (s), 1.37 (s), 1.23 (s) and 1.10 (m) (Table S1). The ^13^ C-NMR displayed signals for 20 carbon resonances including two carbonyl carbons at δ_C_ 202.01 (C-7) and δc 203.93 (C-14), eight olefinic carbons in the region (δc 112.42–183.29), five methyl carbons (δc 6.11–30.40), two methylene carbons at δ_C_ 42.45 (C-1) and 41.23 (C-11), one methyl carbon at δ_C_ 38.34 (C-2) and two quaternary carbons resonating at δ_C_ 36.64 (C-10) and 99.77 (C-15, oxycarbon). The isolated compound was characterized as the macrocyclic diterpenoid jatrophone based on the spectral data (supplementary file 1) and by comparison to the data reported in the literature [35, 36]. The genus *Jatropha* has been found to contain various types of macrocyclic diterpenoids, including jatrophane, rhamnofolane, lathyrane, daphnane, tigliane, dinorditerpene, and pimarane.

### Biological assessment

#### **Cytotoxicity of jatrophone in MCF-7**^**ADR**^**cells**

**Fig. 1:**
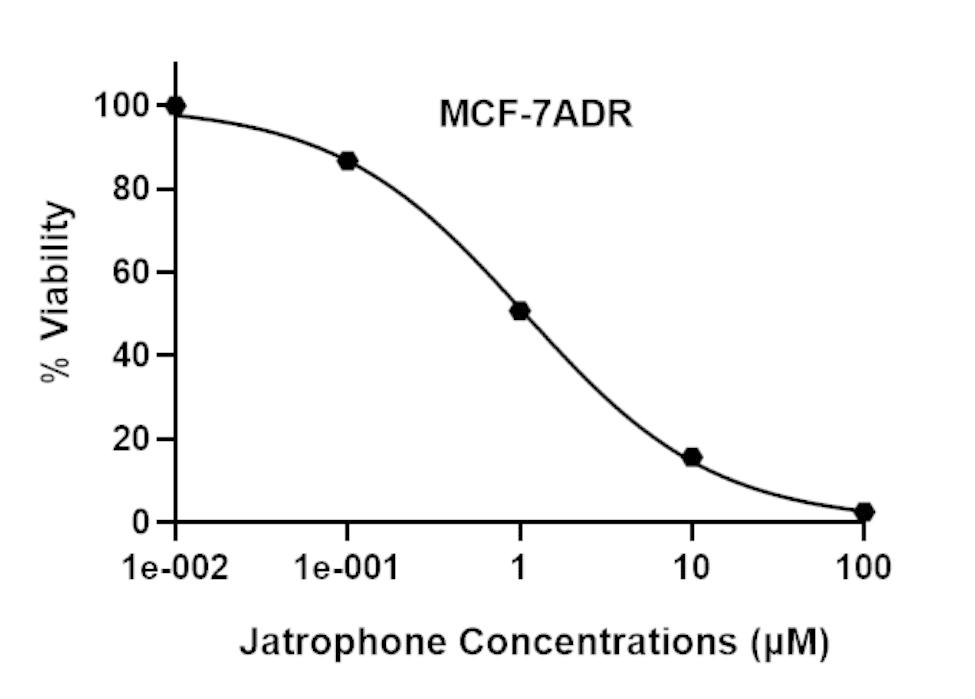
In vitro growth inhibition effect of jatrophone against resistant breast cancer cells (MCF-7^ADR^). Cell viability was evaluated by SRB assay and data are expressed as mean ± SD (n = 3)

Sulforhodamine B (SRB) assay was used to measure the cytotoxicity of jatrophone against MCF-7^ADR^ cells following exposure to doses of 0.1 to 100 µM for 72 h. Cellular log death was progressive in profile and jatrophone exerted gradient cytotoxic activity with increasing concentrations up to 1 M, where viability started to markedly decline with an IC_50_ of 1.8 ± 0.05 µM (Fig. 1).

#### **Effect of jatrophone on the cell cycle in MCF-7**^**ADR**^**cells**

**Fig. 2:**
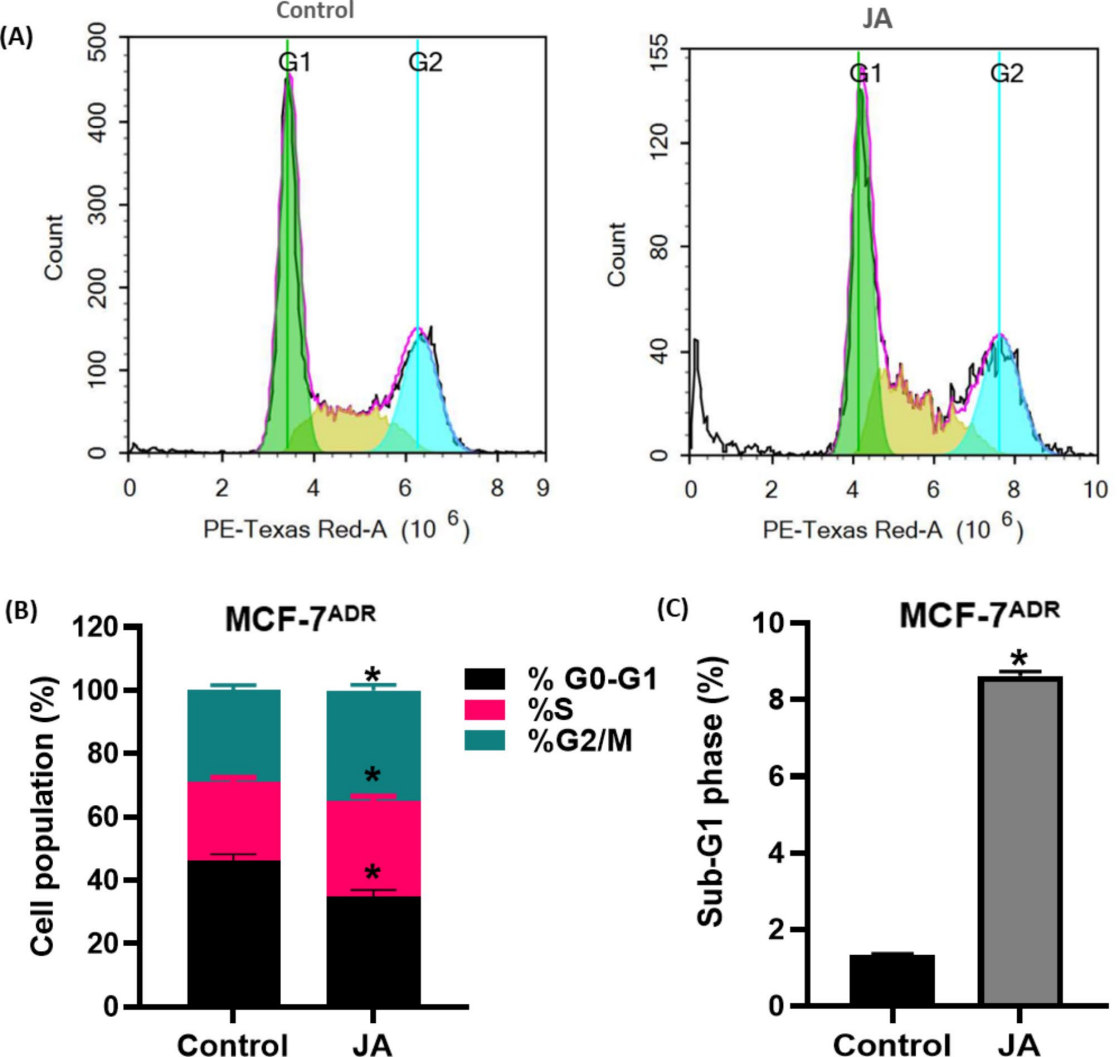
Evaluation of cell cycle progression in resistant breast cancer cells (MCF-7^ADR^) after JA treatment. Cell cycle distribution was determined using DNA cytometry analysis **(A)** of control (untreated cells) and 48 h JA treated MCF-7^**ADR**^ cells and Quantification of percentage **(B)** of cells in each phase of the cell cycle is represented as a bar graph of mean ± SD; n = 3. **(C)** Sub-G1 phase was plotted as percentage of total events. * Statistically significant from control

Cell cycle distribution using DNA flow cytometry was used to assess observed growth inhibition of the MCF-7^**ADR**^ cell line after jatrophone (JA) treatment **(**Fig. 2**).** Jatrophone treatment showed significant changes in all cell cycle phases compared to control cells. JA caused a meaningful decrease in the cell population of G0/G1-phase (the non-proliferating cell proportion) from 46.21 ± 2.7% to 34.83 ± 1.9% compared to control cells. Reciprocally, treatment with JA showed a significant increase in cell percentage of the S phase (the DNA synthesis phase) after 48 h treatment from 24.83 ± 2.01% to 30.17 ± 1.95% concomitantly with an increase of cell percentage in the G2/M phase from 28.96 ± 2.4% to 34.99 ± 1.8% **(**Fig. 2B**)**, indicating cell cycle arrest in two stages and suggesting the difficulty of repairing the intracellular damaged DNA. Accumulation of cells in the G2/M phase constituted enough stress to induce mitotic block and cell death. Correspondingly, JA treatment resulted in a markable cell death manifested by a significant increase in the Sub-G1 phase from 1.35 ± 0.03% to 8.62 ± 0.92% compared to control cells **(**Fig. 2C**)**.

#### Apoptosis/necrosis assessment

To find out the mechanism of cell death (Apoptosis/necrosis) by which MCF-7^**ADR**^ cells underwent cell death induced by treatment with the pre-calculated IC_50_ of jatrophone (JA), annexin-V/FITC double staining coupled with flow cytometry was performed.

**Fig. 3:**
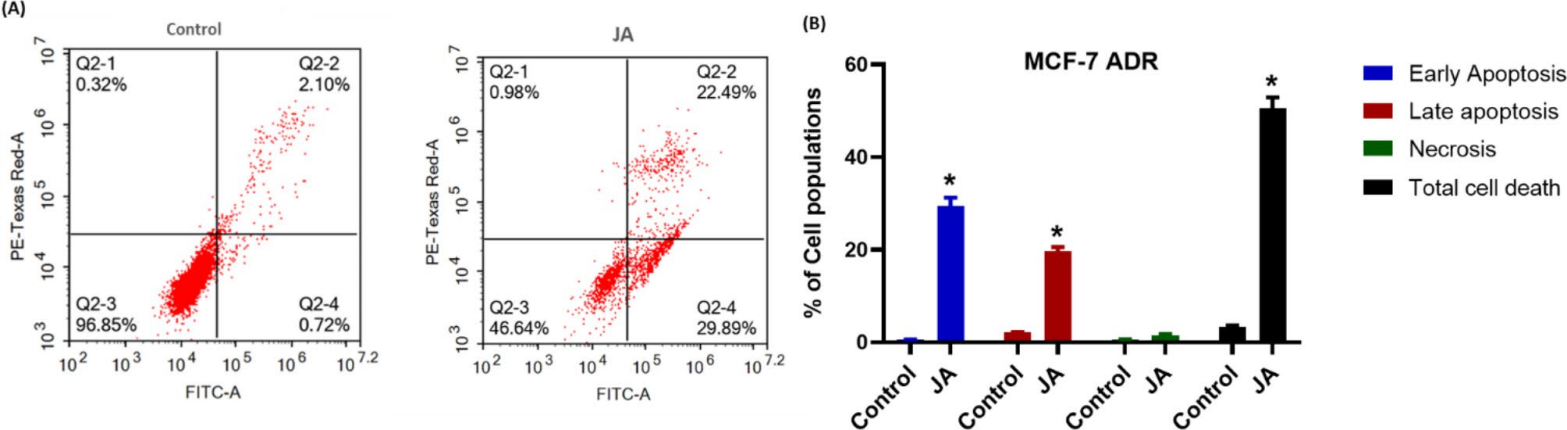
Evaluation of cell death modality (Apoptosis/necrosis) in resistant breast cancer cells (MCF-7^ADR^ cells) after jatrophone treatment for 48 h followed by double staining with Annexin-FITC/PI. (**A**) Quantification of cells in different stages; (**B**) Different cell populations of early apoptosis, late apoptosis, and necrosis with total cell death were plotted as a percentage of total events. Data are presented as mean ± SD; n = 3. * Statistically significant from control

Jatrophone produced profound cell death of resistant breast cancer cells with ~ 52% total cell death, about 30% through early apoptosis, and nearly 22% by late apoptosis **(**Fig. 3A**)**. Interestingly, JA produced induced marked early apoptosis (29.89 ± 2.1%) and late apoptosis (22.49 ± 1.9%) after 48 h of exposure compared to control untreated cells (0.72 ± 0.04%) and (2.1 ± 0.22%), respectively. Though, treatment with JA didn’t induce notable necrotic cell death compared to control cells (0.32 ± 0.01% and 0.98 ± 0.11%, respectively) **(**Fig. 3B**).** These findings showed that resistant breast cancer cells surrendered to cell death only by apoptotic mode, suggesting a prominent role of apoptosis in jatrophone-mediated cell death.

#### **Induction of autophagy by jatrophone in MCF-7**^**ADR**^**cells**

**Fig. 4:**
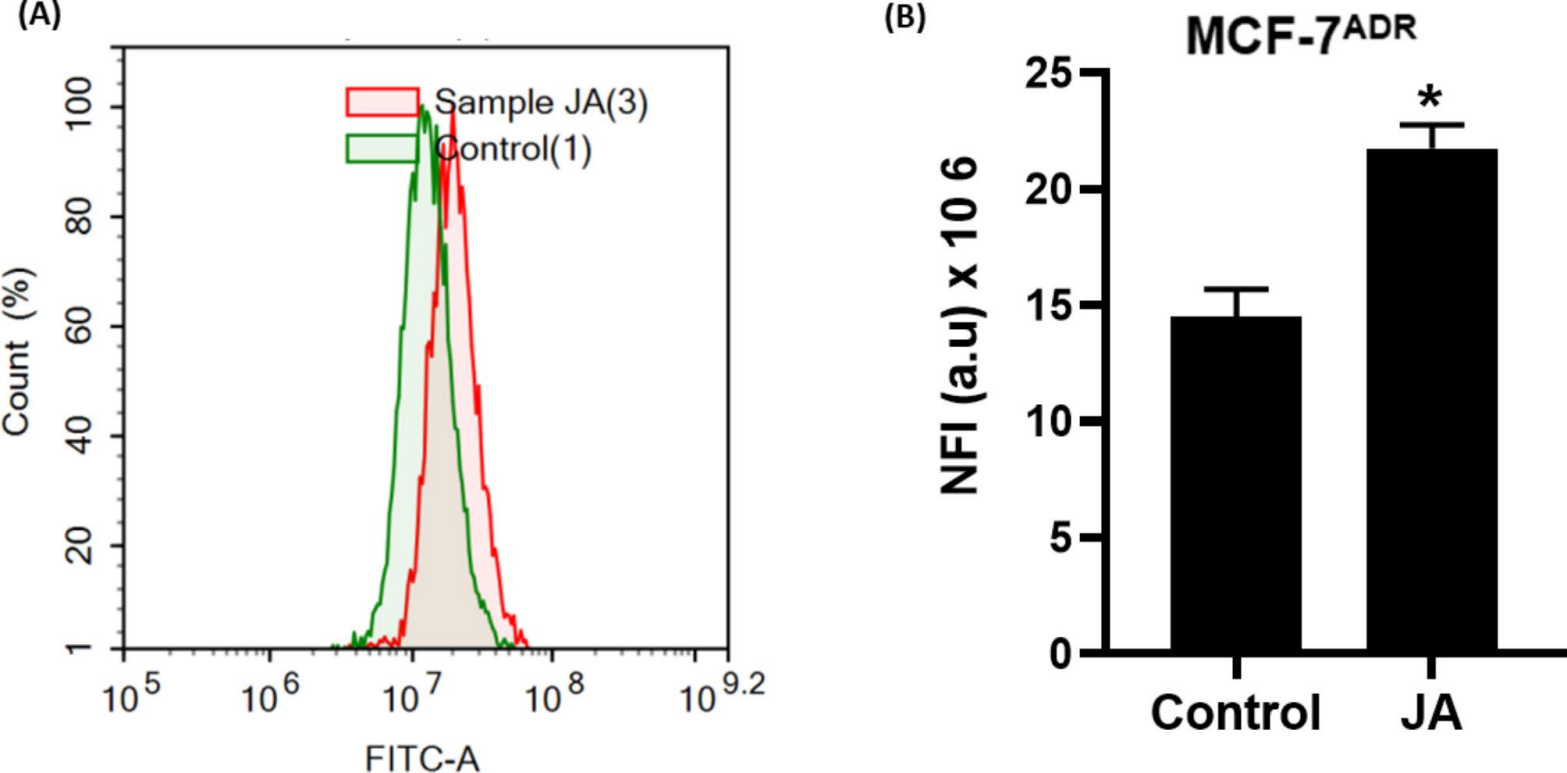
**(A)** Autophagic cell death evaluation in MCF-7^ADR^ cells after exposure to jatrophone for 48 h. Cells were stained with acridine orang dye; **(B)** Net fluorescent intensity (NFI) was plotted in comparison with the basal fluorescence of untreated MCF-7^ADR^ cells; Data are displayed in triplicate. * Statistically significant from control

Accumulating evidence has shown the relationship between autophagy and multidrug-resistant tumors. Thus, it is necessary to understand the exact mechanism of drugs on autophagy. Here, MCF-7^**ADR**^ cells were treated with jatrophone for 48 h, and autophagy was assessed using acridine orange dye coupled with flow cytometry **(**Fig. 4A**)**. The results showed that resistant breast cancer cells showed marked induction of autophagic signal by nearly 33% compared with control cells **(**Fig. 4b**)**. These results suggested that jatrophone at IC_50_ concentration-induced autophagy in resistant breast cancer cells.

#### **Jatrophone inhibits the migration of MCF-7**^**ADR**^**cells**

**Fig. 5:**
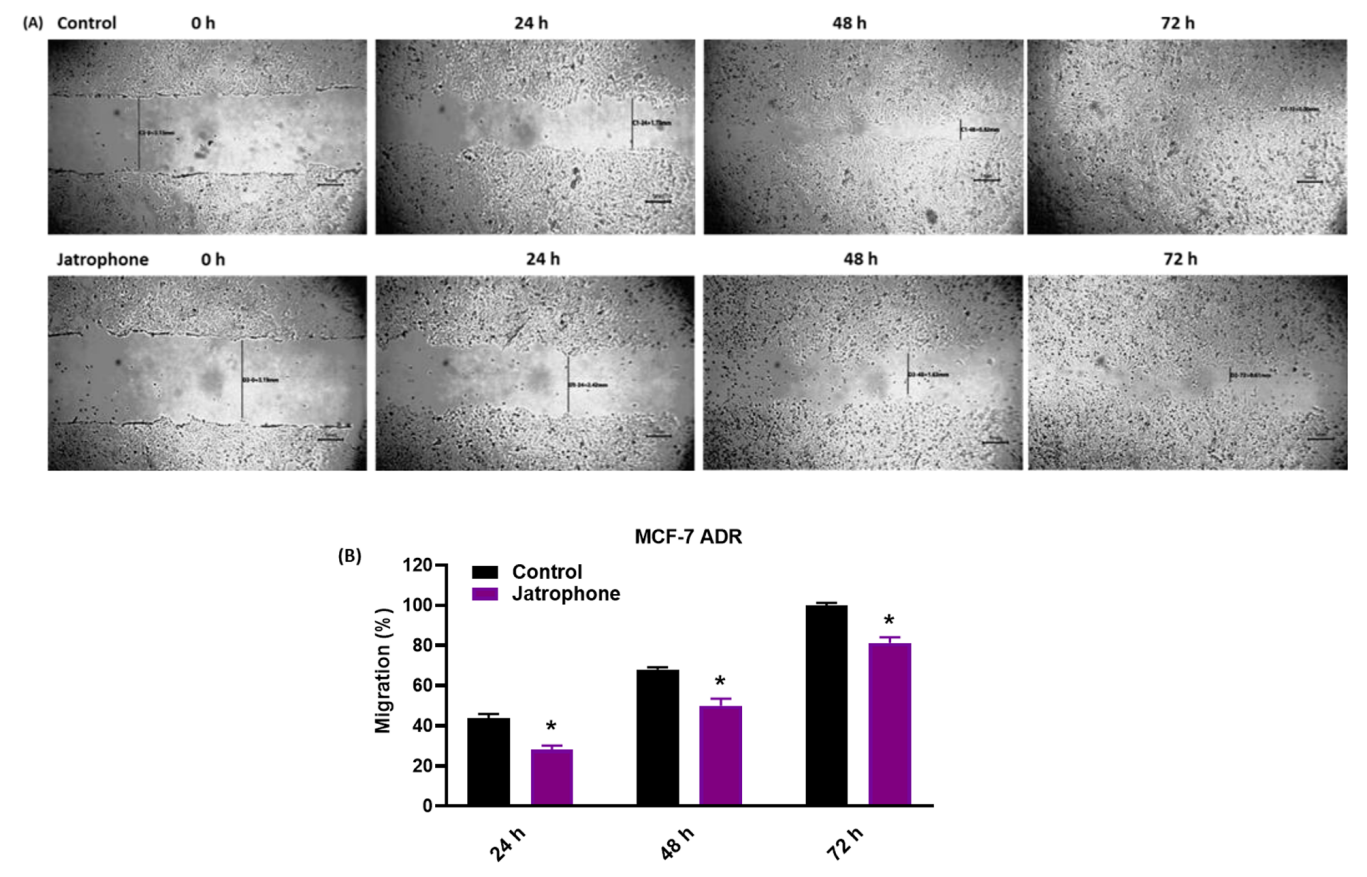
Jatrophone inhibits cell migration of MCF-7^ADR^cells. (**A**) The migration distances were measured after different treatments daily for 72 h; **(B)** Data were blotted as migration % at each time interval; β Data are presented as triplicates. * Statistically significant from control

The inhibitory potential of jatrophone on cell migration of MCF-7^**ADR**^ cells was inspected by performing a Scratch (wound)-healing assay, a critical process associated with EMT and tumorigenesis. Untreated MCF-7^**ADR**^ cells revealed appreciable migratory potential, with a migration rate of 43%, and 68% at 24 and 48 h and showed complete wound closure at 72 h (100% migration), while migration of cells treated with jatrophone was delayed with a migration rate of 28%, 50% and 80% at 24 h, 48 h, and 72 h, respectively **(**Fig. 5A**).** As shown in Fig. 5B, jatrophone suppressed cell migration in a time-dependent manner. β β These results indicated anti-migratory activity of jatrophone on the resistant breast cancer cells.

#### **Jatrophone inhibits PI3K/AKT/ NF-**κ**B pathway in MCF-7**^**ADR**^**cells**

**Fig. 6:**
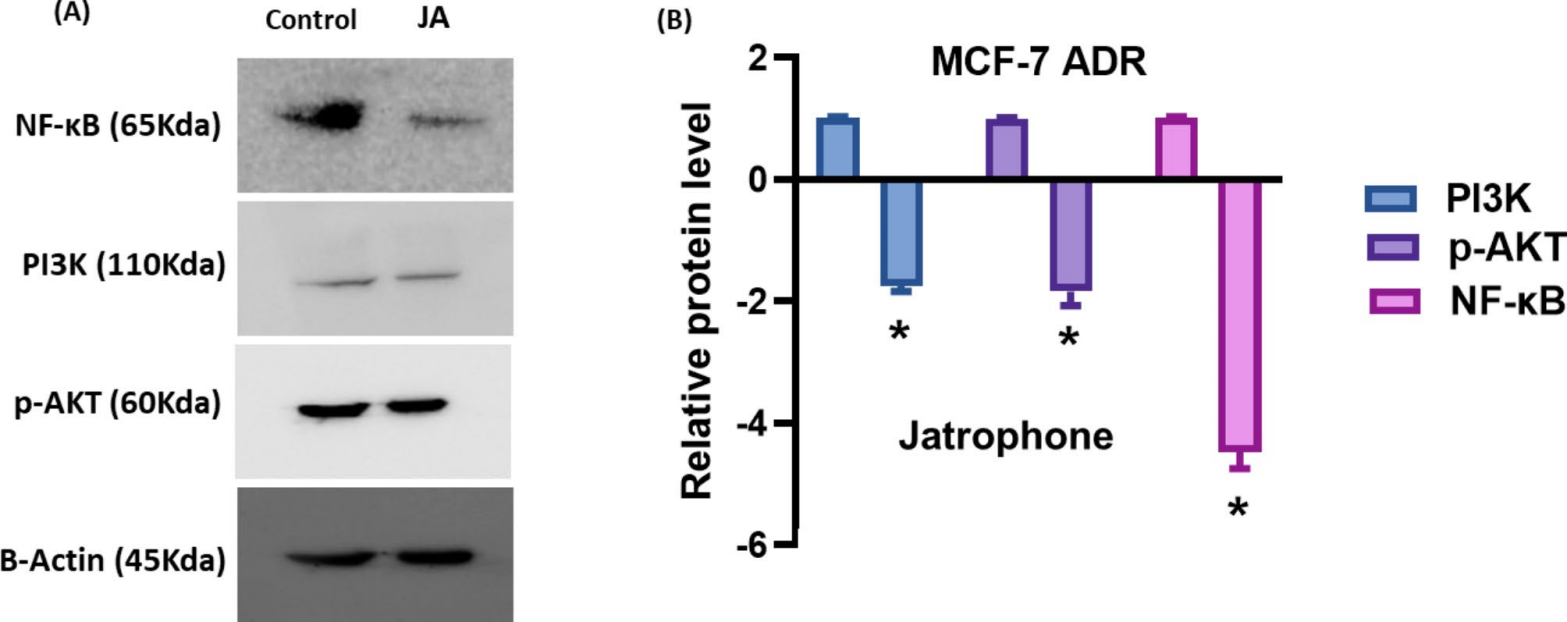
The effect of jatrophone treatment on PI3K/AKT/ NF-κB pathway in MCF-7^ADR^. (**A**) The protein level of PI3K, p-AKT and NF-κB was determined by Western blot analysis in MCF-7^**ADR**^ cells. (**B**) Relative protein levels were represented compared to the control. Data are presented as triplicates. * Statistically significant from control

The PI3K/AKT signaling is a critical pathway involved in breast cancer progression, migration, and resistance development. It also performs a primary function in activating the expression of NF-κB in cancer cells. Thus, we examined the effect of the jatrophone treatment on the expression level of PI3K/AKT and NF-κB cascade. Western blot results showed that jatrophone treatment significantly diminished PI3K, p-AKT, and NF-κB levels compared with untreated cells **(**Fig. 6**)**, suggesting that jatrophone induced anticancer and anti-migratory activities are mediated by inhibition of PI3K/AKT/ NF-κB pathway.

## Discussion

Breast cancer is known for its aggressive and multifactorial nature, particularly in advanced stages, which is attributed to its high propensity for metastasis, relapse, and multidrug resistance. Therefore, there is an urgent need to search for alternative medications from natural products. Currently, plant-based therapeutics have been explored due to their effectiveness with high availability, low cost, and the least possible side effects [37, 38]. So, we aimed in this study to evaluate jatrophone as an effective agent in resistant breast cancer and to gain insight into the underlying molecular mechanism using a doxorubicin-resistant breast cancer cell line MCF-7^ADR^.

Despite the few studies that reported the in vitro antiproliferative activity of jatrophone, its cytotoxicity was potent, and in a concentration-dependent way, displaying IC_50_ values < 10 µM [10, 13]. Herein, we demonstrated for the first time that jatrophone showed potent cytotoxicity against MCF-7^ADR^ resistant breast cancer cells with an IC_50_ value of 1.8 ± 0.2 µM.

The cell cycle is the fundamental process that regulates a cell’s life activities, and its disturbance is one of the most significant mechanisms underlying breast cancer. This process is controlled by signaling pathways, including PI3K/AKT signaling [1]. Moreover, cell cycle arrest induced by chemotherapy was found to be involved in deciding the cancer response to different treatments, whether to increase the sensitivity of cells to treatment or serve as a protective mechanism [39]. Jatrophone caused cell cycle arrest of cancer cells in various stages depending on the kind of cancer in the majority of trials, and this was generally seen together with the activation of apoptosis [14, 15].

In our study, we discovered that jatrophone arrested drug-resistant breast cancer cells in the S and G2/M phases, along with an increase in cells in the pre-G1 phase, which led to cell death [40]. This suggests that jatrophone’s induction of cell cycle arrest overcomes drug resistance rather than serving as a defense mechanism because resistance cells frequently escape alternative cell death when the cell cycle is blocked. Furthermore, jatrophone’s interference with the S-G2/M transition in resistant breast cancer cells, which caused an accumulation of cells to enter the mitotic phase, created enough stress to cause cell death *via* a process known as mitotic catastrophe [41].

Apoptosis, necrosis, and autophagy are three types of programmed cell death that oppose proliferation cellular phenomena and are involved in normal development [42]. The occurrence and development of tumors are believed to be related to blocked apoptosis [43] and abnormal proliferation [6], considering that for the majority of malignancies, tumor cells must acquire the capacity of overwhelming apoptosis to allow their survival [44]. Here a minor population of necrotic cells was seen, and apoptosis was most prominent after jatrophone treatment suggesting JA-induced cell death mainly *via* apoptosis, the most promising mode of cell death for cancer therapy [6, 38].

Accumulating data indicates the intricate association between apoptosis and autophagy in response to various anticancer treatments [45–47]. The evidence suggests that the induction of autophagy/apoptosis, either dependently or independently is a new strategy to overcome drug resistance [48] and as a druggable anticancer therapeutic target of naturally derived phytochemicals [49]. Here, jatrophone added to apoptosis-recruitment, induced autophagic cell death in resistant breast cancer cells This study indicates the potential of jatrophone in preventing the progression of resistant breast cancer cells by triggering apoptotic or autophagic cell death, the main goals of tumor chemoprevention [45, 47, 50].

In breast cancer, epithelial-mesenchymal transition (EMT) is crucial for augmented disease repetition, increased tumor fierceness [3, 17], and anticancer drug resistance [18]. In the current exploration, we found that jatrophone has anti-metastatic potentials on MCF-7 ADR cells, confirmed by scratch assay *β*. *β*.

Additionally, other signaling mechanisms, including the NF-B and PI3K/AKT pathways, continue to regulate EMT [51]. One of the most frequently active signal transduction pathways, the PI3K/AKT signaling pathway is implicated in the proliferation, metastasis, and treatment resistance of breast cancer cells [3, 17, 52], as well as being one of the most frequently activated signal transduction pathways [2]. Therefore, inhibiting the PI3K/AKT signaling pathway is considered a promising strategy for cancer treatment. Several lines of evidence have suggested suppressing AKT would result in cell cycle arrest and death [53, 54], which induce apoptosis and reverse DOX resistance [6, 55]. Our findings demonstrate that jatrophone has anti-EMT and anti-migratory potentials of jatrophone in human-resistant breast cancer cells, likely through its inhibition of the PI3K/AKT pathway. These finding provide a new perspective on the potential use of jatrophone as a metastasis and EMT inhibitor in breast cancer.

The NF-κB has a pivotal role as a mediator for various survival signaling pathways associated with the survival and growth of cancer cells [56], suggesting that jatrophone may exhibit an anti-proliferative effect on resistant breast cancer cells by targeting NF-κB expression. Moreover, this study implied the involvement of NF-kB as a molecular target of jatrophone [14]. Additionally, studies displayed NF-κB as a transcription factor implicated in the phosphoinositide-3-kinase PI3K/AKT signaling pathway in breast cancer [6, 57]. These results indicated that Jatrophone could be a powerful new chemotherapeutic agent against resistant breast cancers by targeting the oncogenic PI3k/AKT/NFkb signaling pathway.

## Conclusion

Considering this study, our data strongly imply that jatrophone **(1)** decreases the proliferation of MCF-7/ADR cells at a low micromolar concentration, **(2)** induces cell cycle arrest, **(3)** promotes apoptotic and autophagic cell death, **(4)** inhibits migration and EMT **(5)** works on resistance by a mechanism involving the inhibition of the PI3K/Akt/ NF-κB pathway. These findings provide evidence to develop jatrophone as a lead molecule for further in vivo and clinical investigation to combat resistant breast cancer.

### Electronic supplementary material

Below is the link to the electronic supplementary material.

Supplementary Material 1

## Data Availability

All data generated or analyzed during this study are included in this published article and its supplementary information file.
