# Supplementary material for: Jatrophone: a cytotoxic macrocylic diterpene targeting PI3K/AKT/NF-κB pathway, inducing apoptosis and autophagy in resistant breast cancer cells
Source: BMC Complement Med Ther. 2023 Aug 22;23:293. doi: 10.1186/s12906-023-04113-6 (PMC10463460; doi:10.1186/s12906-023-04113-6)
Supplement: Supplementary file 1 — Supplementary Material 1 [file 12906_2023_4113_MOESM1_ESM.docx]

**Supplementary materials**

**Jatrophone: A Cytotoxic Macrocylic Diterpene Targeting PI3K/AKT/NF-κB Pathway, Inducing Apoptosis and Autophagy**

Khawlah Shari^1^, Rania A. El Gedaily^1^, Rasha M. Allam^2^, Khaled M. Meselhy^1^, Amal E. Khaleel^1^, Essam Abdel-Sattar^1, *^

^1^Pharmacognosy Department, Faculty of Pharmacy, Cairo University, Kasr El-Aini St., 11562 Cairo, Egypt; ^2^Pharmacology Department, Medical Research Institute, National, Research Centre, 12622, Dokki, Cairo, Egypt;

*****Corresponding author

Essam Abdel-Sattar

essam.abdelsattar@pharma.cu.edu.eg; Tel.: [+2023639307](Tel:23639307); Fax: +2023628426,

Cell phone: +201065847211

**Content**

**Table S1. Spectral data of jatrophone**

**Figure S1. ^1^H-NMR spectrum of jatrophone in CDCl_3_**

**Figure S2. ^13^C-NMR spectrum of jatrophone in CDCl_3_**

**Figure S3: H-H COSY spectrum of jatrophone in CDCl_3_**

**Figure S4: HMQC spectrum of jatrophone in CDCl_3_**

**Figure S5. HMBC spectrum of jatrophone in CDCl_3_**

**Figure S6. Western blot of Beclin-1 (autophagy marker), β‐catenin (EMT‐related protein) and β‐actin (Housekeeping protein) in MCF-7^ADR^ cells**

**Figure S7. Western blot of PI3K, NFK-b and p-Akt and β‐actin (Housekeeping protein) in MCF-7^ADR^ cells**

**Table S1. Spectral data of jatrophone (400 MHz for ^1^H- and 100 MHz for ^13^C-NMR) in CDCl_3_.**

| **No.** | **^1^H-NMR** | **^13^C-NMR** |
| --- | --- | --- |
| 1 | 1.86 (dd, 7.9, 13.8), 2.15 (*dd*, 5.8, 13.8) | 42.45 |
| 2 | 2.98 (q, 6.9) | 38.33 |
| 3 | 5.80 (m) | 141.77 |
| 4 | - | 137.10 |
| 5 | 5.78 (m) | 123.74 |
| 6 | - | 147.11 |
| 7 | - | 202.01 |
| 8 | 5.99 (d, 16.0) | 128.71 |
| 9 | 6.44 (d, 16.0) | 159.06 |
| 10 | - | 36.64 |
| 11 | 2.86 (d, 15.0), 2.40 (d, 15.0) | 41.22 |
| 12 | - | 183.29 |
| 13 | - | 112.42 |
| 14 | - | 203.93 |
| 15 | - | 99.77 |
| 16 | 1.08 (d, 7.1) | 18.98 |
| 17 | 1.87 (d, 1.55) | 20.75 |
| 18 | 1.23 (s) | 30.40 |
| 19 | 1.35 (s) | 26.91 |
| 20 | 1.74 (s) | 6.11 |


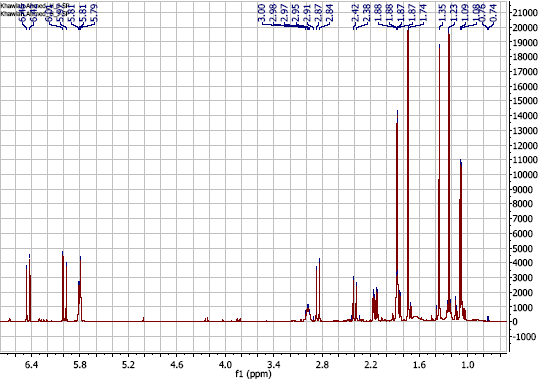


**Figure S1. ^1^H-NMR spectrum of jatrophone in CDCl_3_**


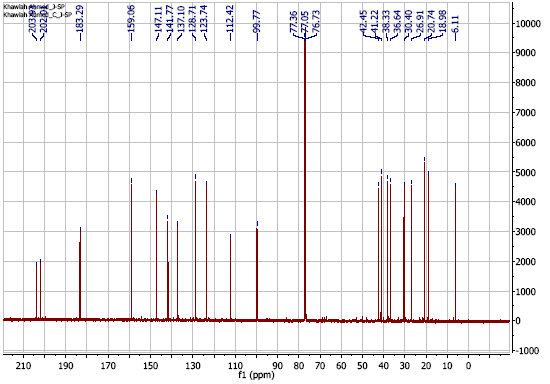


**Figure S2. ^13^C-NMR spectrum of jatrophone in CDCl_3_**


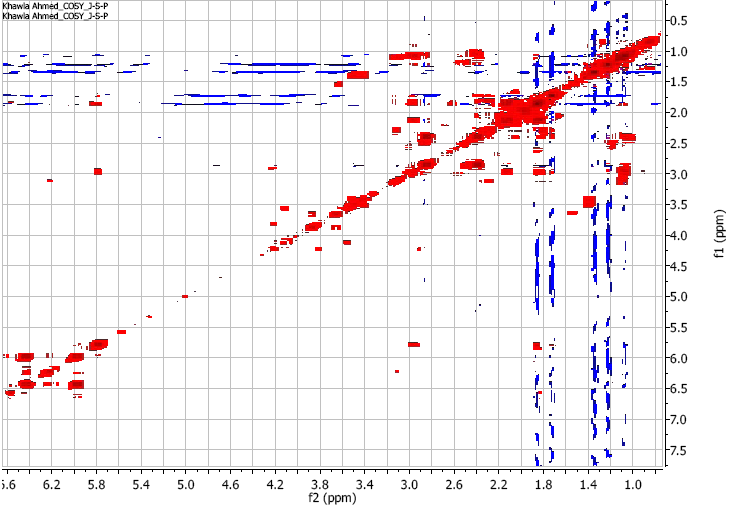


**Figure S3: H-H COSY spectrum of jatrophone in CDCl_3_**


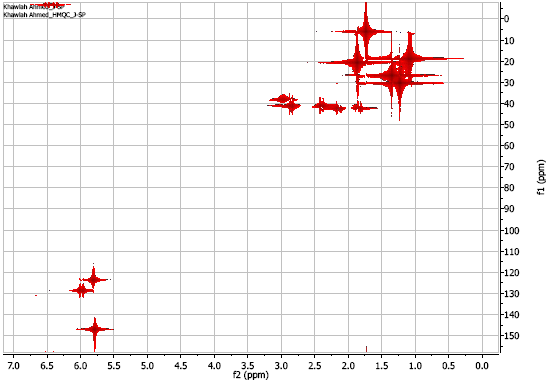


**Figure S4: HMQC spectrum of jatrophone in CDCl_3_**

**Figure S5. HMBC spectrum of jatrophone in CDCl_3_**


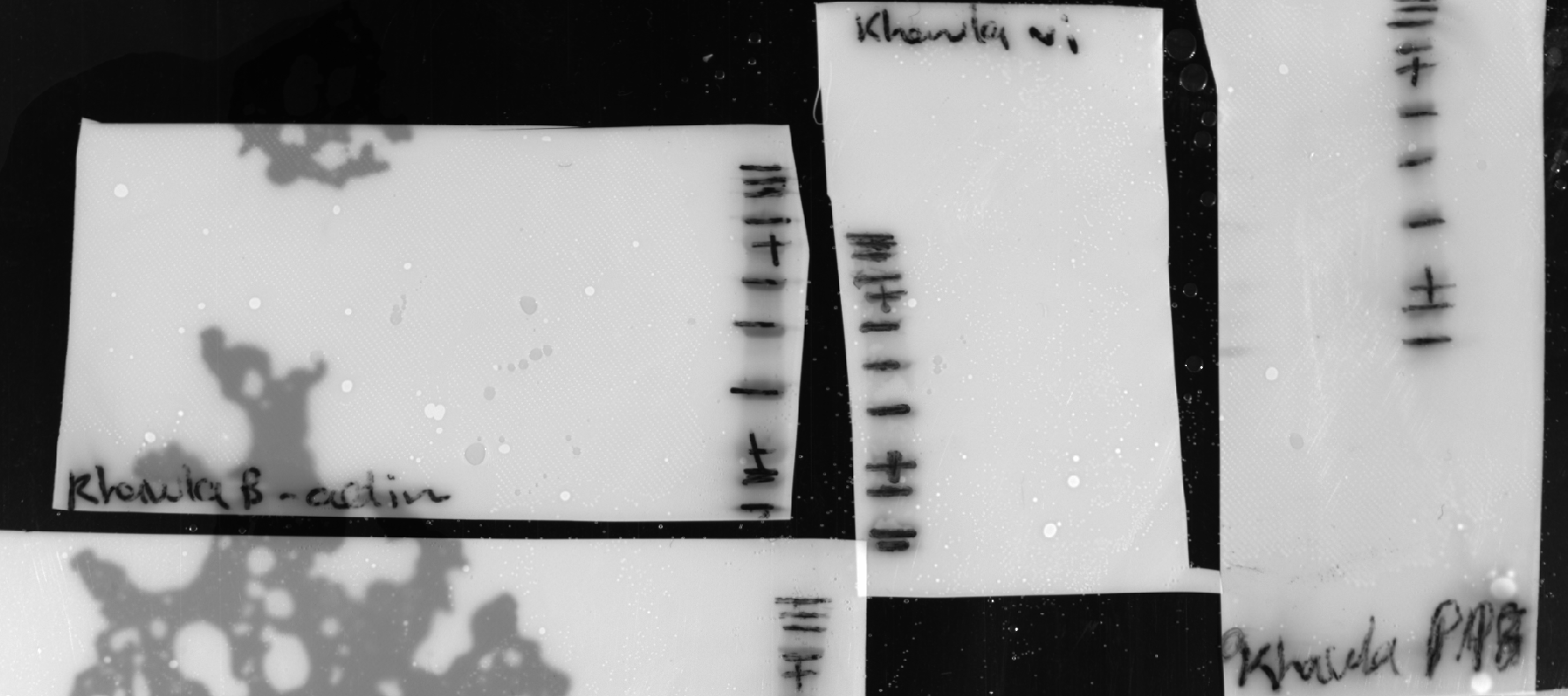

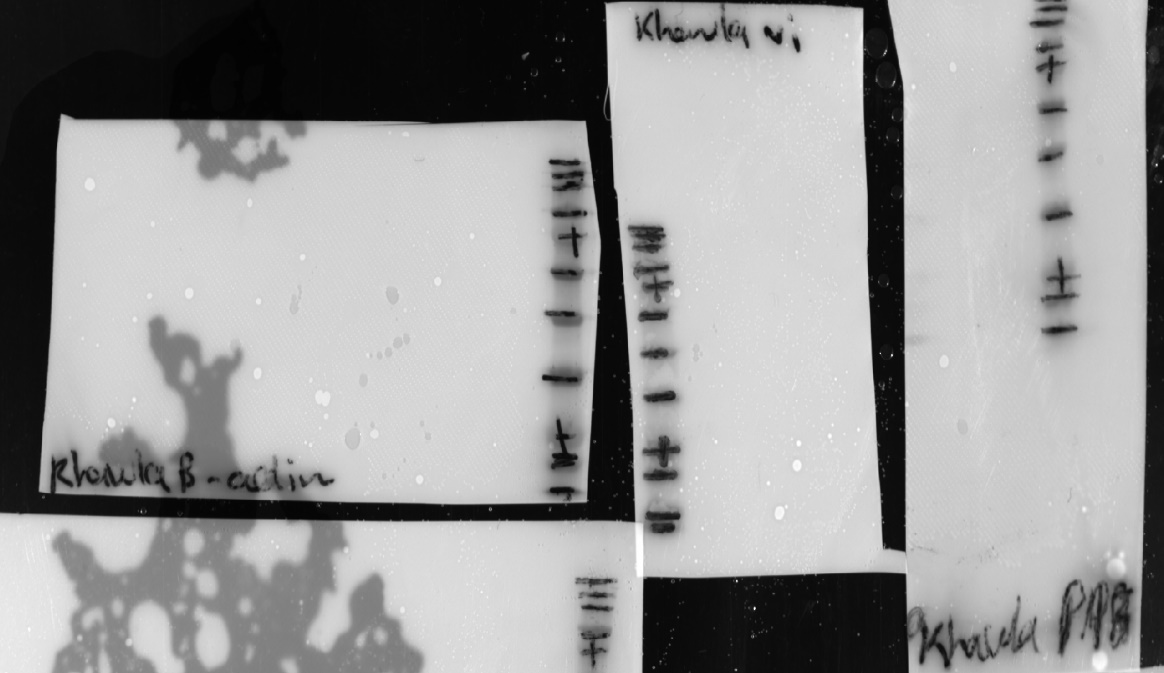

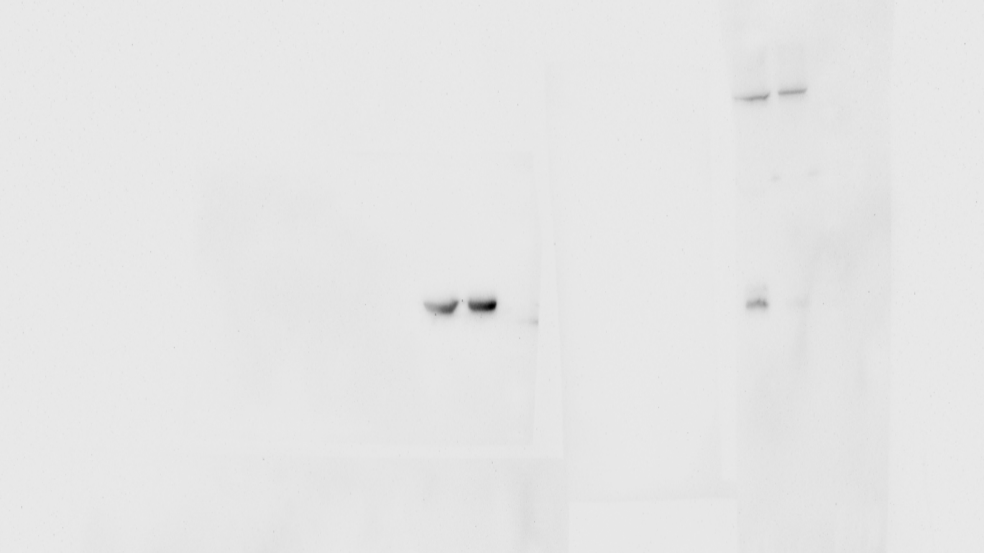

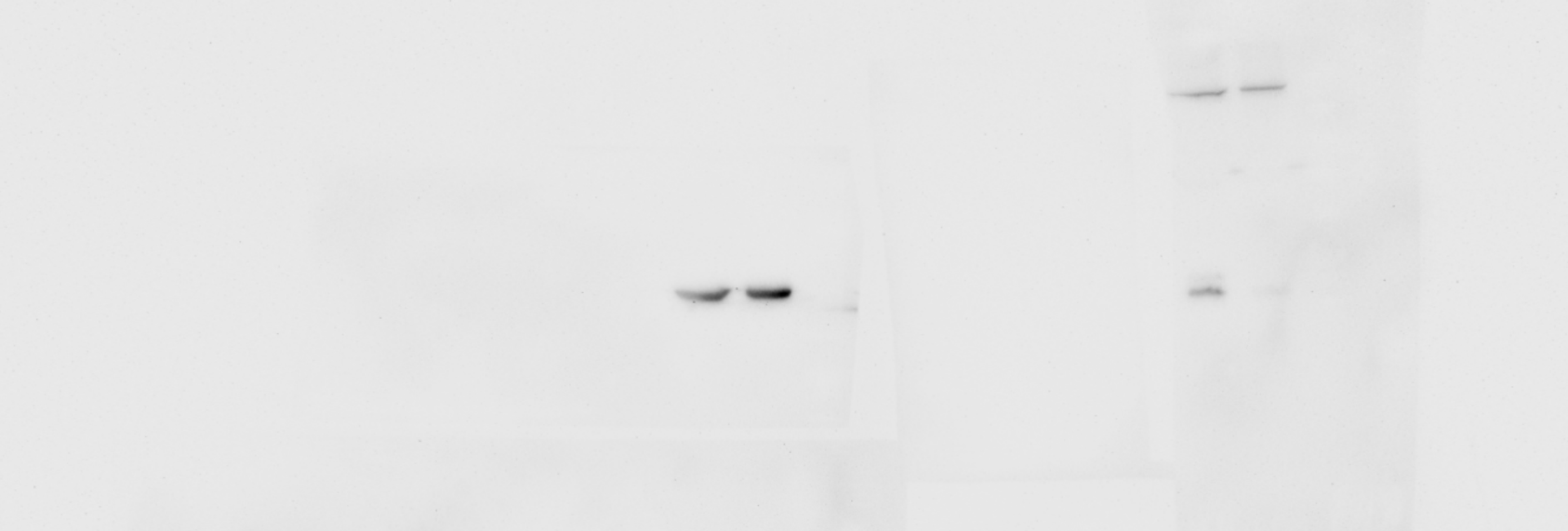

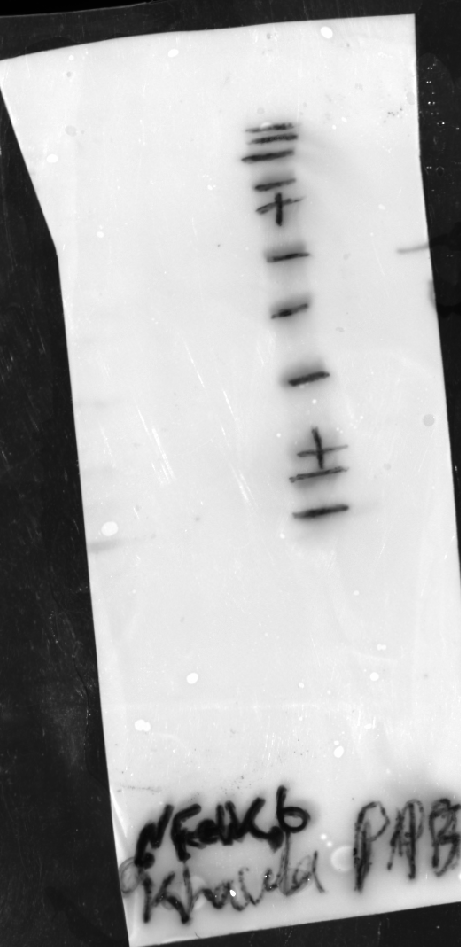

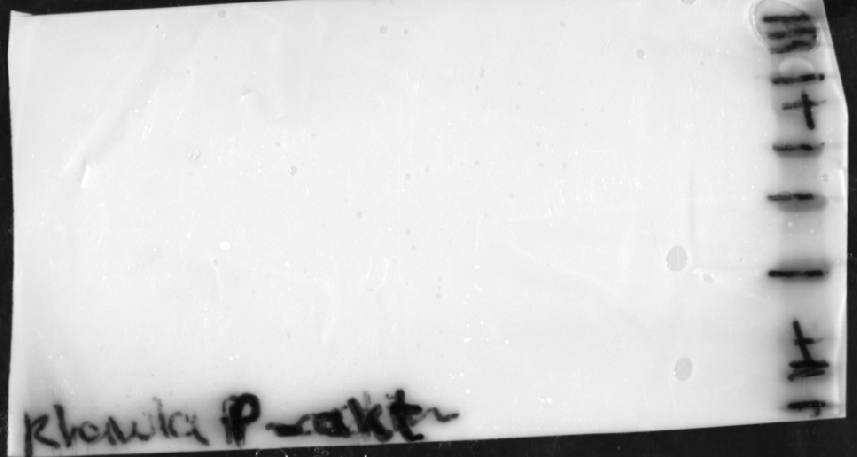

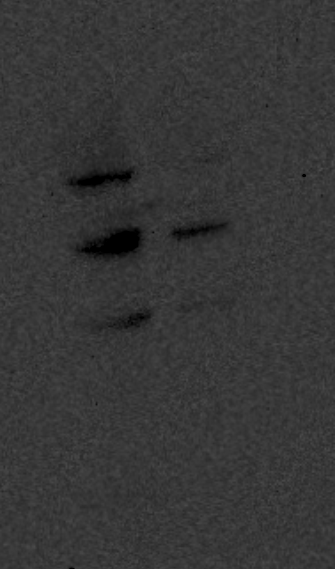

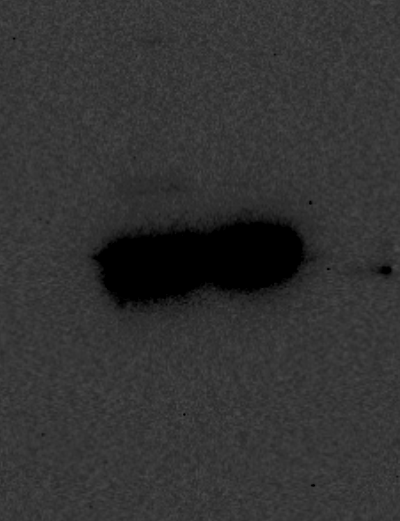


PI3K

B- actin

NFK-b

P-AKT

**Figure S6.** Western blot of PI3K, NFK-b and p-Akt and β‐actin (Housekeeping protein) in MCF-7^ADR^ cells


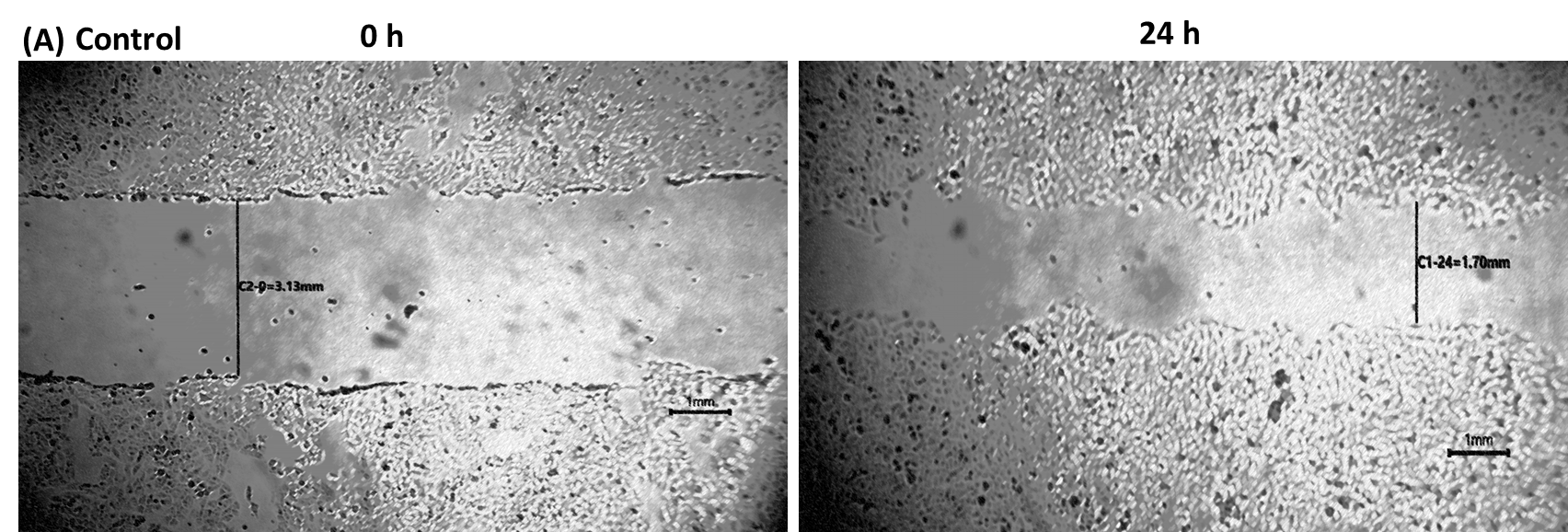


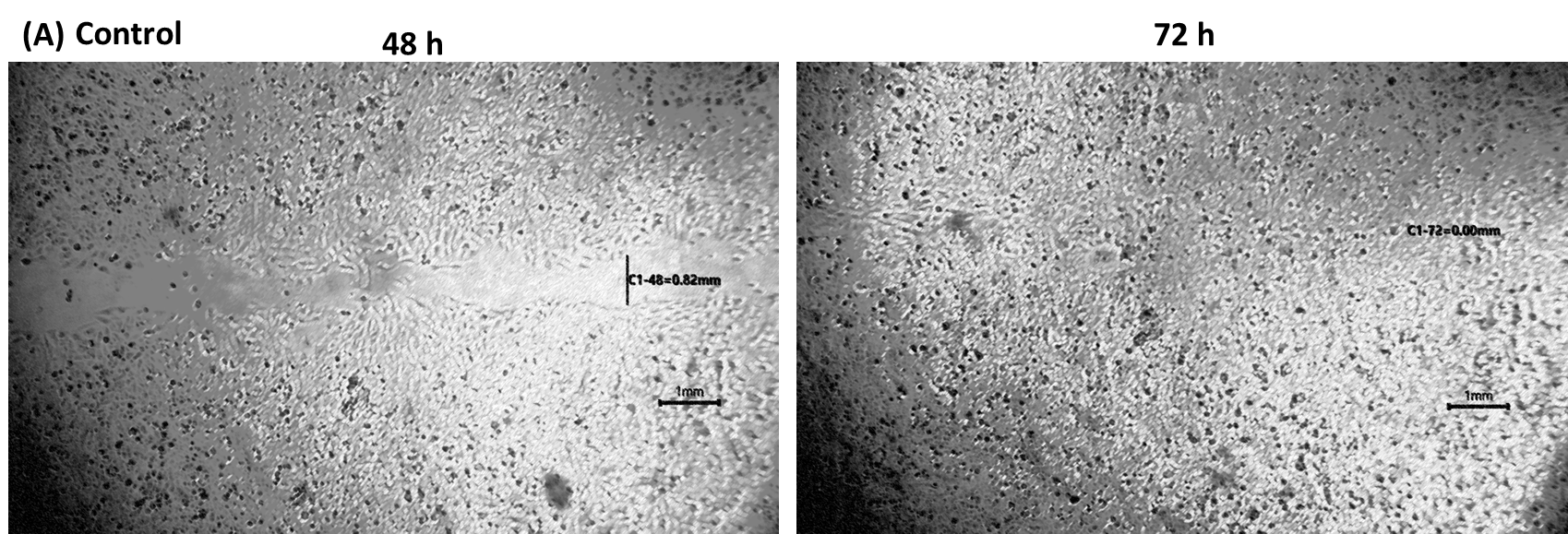


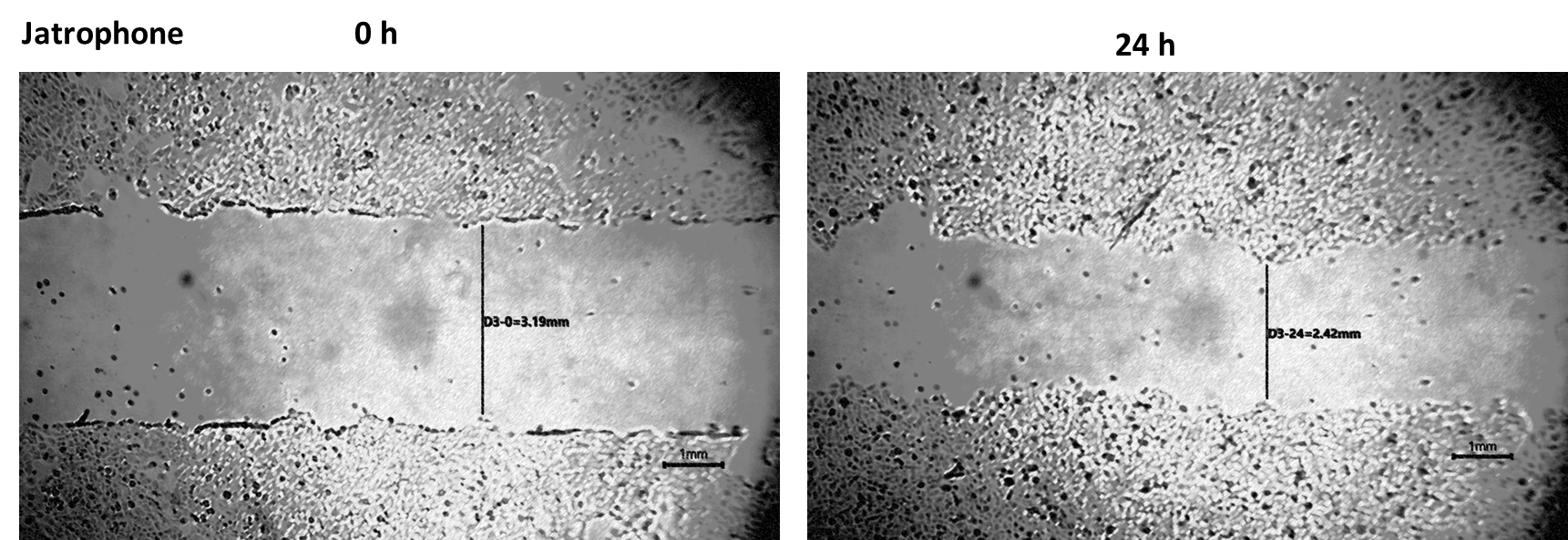


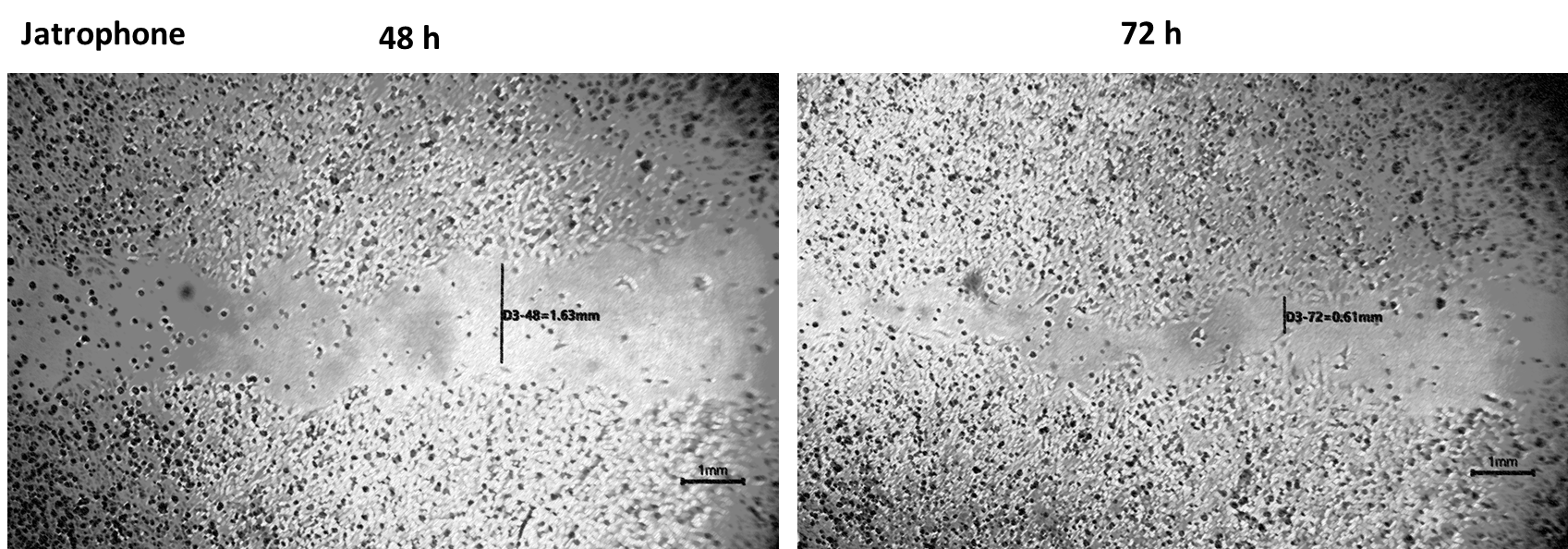


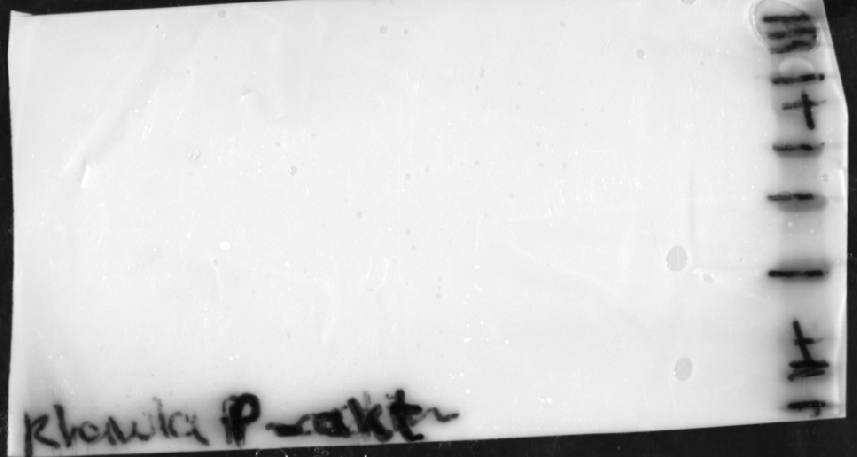
Figure S7. The migration distances of control and jatrophone-treated MCF-7**^ADR^** cells at 0, 24, 48, and 72h.

PI3k

B- actin

NFKb

P-AKT


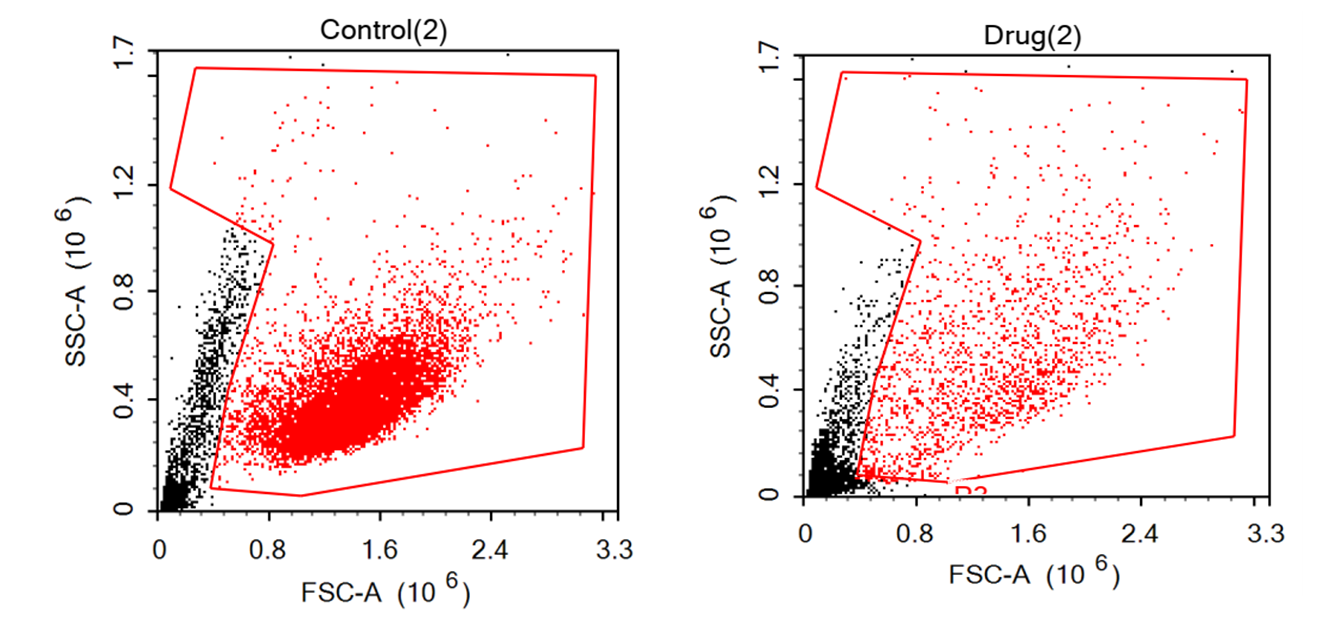


Figure S8. Raw data of flow cytometry showing the applied gating.
